# Preoperative planning and intraoperative real-time navigation with indocyanine green fluorescence in robotic liver surgery

**DOI:** 10.1007/s00423-023-03024-x

**Published:** 2023-07-31

**Authors:** Gianluca Rompianesi, Francesca Pegoraro, Lorenzo Ramaci, Carlo DL Ceresa, Roberto Montalti, Roberto I Troisi

**Affiliations:** 1 Division of Hepato-Biliary-Pancreatic, Minimally Invasive and Robotic Surgery and Kidney Transplantation, Department of Clinical Medicine and Surgery, Federico II University Hospital, via S. Pansini n. 5, Naples, Italy; 2 Nuffield Department of Surgical Sciences, University of Oxford, Oxford, UK; 3 Division of Hepato-Biliary-Pancreatic, Minimally Invasive and Robotic Surgery and Kidney Transplantation, Department of Public Health Federico II University Hospital, Naples, Italy

**Keywords:** Hepatobiliary surgery, Robotic surgery, Robotic liver resection, Indocyanine green, Intraoperative fluorescence, Preoperative planning

## Abstract

**Purpose:**

We aimed at exploring indocyanine green (ICG) fluorescence wide spectrum of applications in hepatobiliary surgery as can result particularly useful in robotic liver resections (RLR) in order to overcome some technical limitations, increasing safety, and efficacy.

**Methods:**

We describe our experience of 76 RLR performed between March 2020 and December 2022 exploring all the possible applications of pre- and intraoperative ICG administration.

**Results:**

Hepatocellular carcinoma and colorectal liver metastases were the most common indications for RLR (34.2% and 26.7% of patients, respectively), and 51.3% of cases were complex resections with high IWATE difficulty scores. ICG was administered preoperatively in 61 patients (80.3%), intraoperatively in 42 patients (55.3%) and in both contexts in 25 patients (32.9%), with no observed adverse events. The most frequent ICG goal was to achieve tumor enhancement (59 patients, 77.6%), with a success rate of 94.9% and the detection of 3 additional malignant lesions. ICG facilitated evaluation of the resection margin for residual tumor and perfusion adequacy in 33.9% and 32.9% of cases, respectively, mandating a resection enlargement in 7.9% of patients. ICG fluorescence allowed the identification of the transection plane through negative staining in the 25% of cases. Vascular and biliary structures were visualized in 21.1% and 9.2% of patients, with a success rate of 81.3% and 85.7%, respectively.

**Conclusion:**

RLR can benefit from the routine integration of ICG fluoresce evaluation according to each individual patient and condition-specific goals and issues, allowing liver functional assessment, anatomical and vascular evaluation, tumor detection, and resection margins assessment.

## Introduction

Hepatobiliary surgery continues to develop as a specialty and its most recent advances have majorly benefited from the constant introduction of innovative technologies [1–4]. Above all, minimally invasive surgery, initially restricted by limited expertise and available devices, has witnessed an explosive growth in the last decade [3, 5]. As the latest addition to the surgeon’s armamentarium, robotic systems such as the Da Vinci® platform are in steady expansion and show promising results in terms of safety and feasibility even in the most complex cases [6–9]. The latest technological developments are aimed at overcoming some of the technical limitations, such as lack of haptic feedback, difficult handling and change of some instruments, and the unavailability of some devices (such as the cavitron ultrasonic surgical aspirator) [1, 6, 8, 10]. Its integration with other operative adjuncts, such as intraoperative ultrasound (IOUS), 3D organ and vascular modeling, and indocyanine green (ICG) fluorescence, has progressively developed and can partially compensate for some of the robot’s shortcomings; by providing an important contribution through aid and assistance, especially in the most challenging settings [11–13]. ICG is an inactive substance exclusively excreted by the liver into the biliary tract, which has been adopted for several applications over the decades. This is particularly true in hepatobiliary surgery, where the use of ICG has becoming increasingly more common [14, 15]. Thanks to its affordability and high safety profile, ICG has undergone a rapid expansion of its indications and applications for benign and malignant conditions in both the preoperative and intraoperative settings [14–20]. To achieve the integration and real-time utilization of the robotic surgical system with the available diagnostic tools, the Xi version of the Da Vinci® robotic system allows intraoperative ICG fluorescence visualization through the Firefly mode™, which can be activated directly from the console by the operating surgeon [21, 22]. This modality results in a minimal workflow disruption during surgical maneuvers and can be further merged with other imaging without device or monitor adjustments.

This manuscript describes our experience of 61 consecutive robotic hepatic resections (RLR) performed with the aid of pre- and intraoperative ICG fluorescence and reviewing all the possible applications of ICG fluorescence in robotic hepatobiliary surgery.

## Methods

Data from all consecutive patients undergoing RLR with pre- and/or intraoperative ICG fluorescence at our Institution between March 2020 and December 2022 were collected in a prospective database and enrolled in this study. Patients affected both by malignant and benign diseases were included. Cholecystectomies, cysts fenestrations, and thermal ablations were excluded.

The indication for performing a robotic approach was not subject to restrictions concerning previous abdominal surgery or the extent/complexity of the planned hepatic resection. However, it is our standard practice to treat patients that underwent two or more previous hepatic resections with a laparoscopic rather than robotic technique due to the possible presence of severe adhesions leading to a higher chance of open conversion, especially if the previous resections were performed via an open approach.

As per ICG contra-indications, only patients with hyperthyroidism and iodine intolerance were excluded from its administration.

The preoperative work-up routinely included complete blood tests, with serum tumor markers’ evaluation if appropriate, and a thorough physical examination with chest x-ray, electrocardiogram, and an anesthetic assessment. Depending on the primary disease, a whole-body contrast-enhanced computed tomography (CT) and/or a liver gadoxetic acid–enhanced magnetic resonance imaging (MRI) was performed. Positron-emission tomography (PET) and contrast-enhanced abdominal ultrasound (CEUS) were performed selectively in specific cases only when indicated. For all patients, the surgical indications were defined at the institutional multidisciplinary meeting discussion.

Preoperative data were collected to register sex, age, body mass index (BMI), liver function (Child-Pugh and model for end-stage liver disease (MELD) scores), general performance status (Eastern Cooperative Oncology Group (ECOG) and Karnofsky sores), viral infections, comorbidities, alcohol intake, American Society of Anesthesiologists (ASA) score [23], Charlson comorbidity index (CCI) [24], previous abdominal and liver-directed therapies, preoperative diagnosis, neoadjuvant chemotherapy, main preoperative blood chemistry values (platelets, international normalized ratio (INR), total serum bilirubin, albumin, creatinine) and indications for surgery. Each procedure was assigned a difficulty score according to the IWATE score [25].

The following perioperative outcomes were analyzed: the type and extent of liver resection (according to the Tokyo 2020 terminology) [26], the route, timing, and dose of ICG administration, the accuracy of ICG fluorescence in identifying the liver lesions and its concordance with the histopathology report, the open conversion rate, the execution and duration of the Pringle maneuver, the intraoperative blood loss, the perioperative transfusion rate, the operative time, length of stay, the intra- and postoperative complications within 90 days from surgery (based on Clavien-Dindo and Comprehensive Complication Index classifications) [27, 28], the perioperative mortality, and the histologic characteristics of the lesions and the liver parenchyma. The malignant lesions’ grading was determined according to the Edmondson score and the resection margins were defined as negative (R0) when the distance from the tumor was ≥1 mm, or as positive (R1) when <1 mm. Post-hepatectomy liver failure (PHLF) and bile leakage were defined according to the International Study Group of Liver Surgery (ISGLS) definitions [29, 30].

For all complex cases, a preoperative triphasic CT scan was obtained (with 1- or 2-mm-thick slices), in order to elaborate the images with 3D Software (Synapse 3D®, Fujifilm, Tokyo, Japan) and to create a virtual three-dimensional model of the patient’s liver. This helped determine the spatial relationship between lesions and intrahepatic structures, surgical strategy, and the viable pedicles for negative/positive staining. In complex cases, various resection hypotheses could be postulated. The models were studied preoperatively by the surgical team and intraoperatively on a dedicated screen in the operating room and in the robotic console. IRB approval and patient written consent was not required.

### Indocyanine green administration

#### Preoperative administration for a liver function assessment test

Prior to a major hepatic resection, or when there are specific concerns regarding diseased liver parenchyma, a functional liver assessment can be performed by evaluating the retention rate at 15 min after intravenous administration of ICG (ICG-R15) and plasmatic disappearance rate (PDR) [20] calculated by performing the LiMON test. Whenever possible, the test should be performed 2–4 days prior to surgery to take advantage of ICG’s lesions-enhancing properties.

#### Preoperative administration for malignant lesions’ enhancement

ICG is administered 2–4 days before surgery [15, 16] at the dose of 0.25–0.50 mg/kg. Timing and doses can be calculated empirically based on patient age, liver parenchyma status, Child-Pugh score, and type of lesion(s). This technique allows the intraoperative visualization of superficial lesions (not deeper than ≈8 mm from the liver surface) [16].

The cut surface (AIM-TEc) can be evaluated to assess the eventual presence of residual tumor at the end of the liver transection. Similarly, an ex situ assessment on the specimen (AIM-TEx) can be performed to evaluate the proximity of the lesion to the surgical margin. In both cases, a residual fluorescence is highly suggestive of R1/R2 margins and mandate a re-resection of the margin [31].

#### Negative staining

The target Glissonean pedicle is identified, isolated, and clamped. The ICG bolus (2.5 mg in 2 mL of injectable solution) is injected either systemically by the anesthetist or in the upstream main portal vein by the surgeon through a small needle. The fluorescence is then used to assess the limits of the clamped segment, sector, or hemi-liver that appear not enhanced and surrounded by fluorescent parenchyma [17, 32]. This aims to accurately perform an anatomical resection.

#### Positive staining

As opposed to the negative staining, the ICG bolus (1.25 mg in 5 mL of injectable solution) is injected directly by the surgeon downstream of the clamped pedicle of interest, resulting in the selective fluorescence of the target parenchyma only [19, 32]. Similar to the negative staining, this technique allows a precise anatomical resection.

#### Vascular and biliary structures’ enhancement

The ICG bolus is administered in a peripheral vein (2.5 mg in 2 mL of injectable solution), and the enhancement time can vary from a few seconds (for arterial and venous structures, AIM-VE) to 45–60 min (for biliary structures, AIM-BE). Arterial vessels tend to be enhanced slightly sooner than portal pedicles, and portal pedicles earlier than hepatic veins. For this reason, it is advisable to fully mobilize the liver and perform the hilar dissection before ICG administration in order to obtain reliable reference landmarks (hepatic artery, main portal vein, vena cava, or hepatic veins). Usually, an interval time of 30–45 min is sufficient to allow an almost complete excretion of ICG from the liver parenchyma and the surrounding tissues, at which point a selective biliary fluorescence is achieved. Alternatively, the cystic duct stump can be cannulated and directly injected with ICG, producing an immediate biliary fluorescence. After liver transection, this technique can be useful to detect biliary leaks, although we prefer to use a white dye (propofol or lipid emulsion) test due to an excellent color contrast which allows immediate visualization and the fact that the white dye can be rapidly washed away to repeat the test.

#### Resection margin vascularization

The ICG bolus (2.5 mg in 2 mL of injectable solution) is administered systemically after completion of the RLR. The aim is to evaluate the presence of non-fluorescent, ischemic parenchyma at the transection margin and consider the necessity of a further resection, as well as hypovascular areas that can lead to biliary leaks, abscesses, and collections.

### Surgical technique and fluorescence visualization

All the operations were performed with a Da Vinci Robotic Xi™ Surgical System (Intuitive Surgical Inc.®, Sunnyvale, CA, USA). Intraoperative fluorescence imaging was obtained through the integrated Firefly mode™, which can be activated directly by the operating surgeon. The principles of the surgical technique have been already described elsewhere [33–37].

The type of resection was preoperatively decided based on the lesions’ extension, their relationship with the liver’s major vascular structures, their nature and the quality of the liver parenchyma, but subject to intraoperative changes in case of unexpected or incidental findings.

During parenchymal transection, a constant IOUS, 3D rendering, and fluorescence monitoring and navigation were performed to identify and follow the most appropriate resection plane.

### Statistical analysis

Categorical data were reported as frequencies and percentages. Continuous variables were reported as mean ± standard deviation (ranges) for normal distributions; non-parametric variables were reported as median and ranges.

Statistical analysis was performed using IBM SPSS Statistics for Windows, version 26.0 (SPSS Inc. Chicago, IL, USA).

## Results

ICG was administered preoperatively in 61 patients (80.3%), intraoperatively in 42 patients (55.3%) and both pre- and intraoperatively in 25 patients (32.9%) (Table 1). No ICG-related adverse events were recorded.

**Table 1:** Indocyanine green administration, employment, and results

ICG employment and results	Robotic liver resections *n*=76
Preoperative ICG, *n* (%)	61 (80.3%)
Intraoperative ICG, *n* (%)	42 (55.3%)
Preoperative and intraoperative ICG, *n* (%)	25 (32.9%)
LiMON test (AIM-LF), *n* (%)	28 (36.8%)
Tumor identification (AIM-TE), *n* (%)	59 (77.6%)
Negative staining (AIM-NS), *n* (%)	19 (25%)
Positive staining (AIM-PS), *n* (%)	7 (9.2%)
Vessels’ identification (AIM-VE), *n* (%)	16 (21.1%)
Biliary tree enhancement (AIM-BE), *n* (%)	7 (9.2%)
Cut surface perfusion (AIM-RM), *n* (%)	25 (32.9%)
Liver resection margin evaluation (AIM-TEc), *n* (%)*	21 (33.9%)
Ex situ specimen assessment (AIM-TEx), *n* (%)*	12 (19.4%)
Number of lesions incidental identified via ICG	5
Resection enlargements, *n* (%)	6 (7.9%)
ICG suspicious for residual tumor, *n* (%)*	4 (6.5%)
Poor margin perfusion, *n* (%)	2 (2.6%)
Resection margin, mm	3 (0–75)
R1*, *n* (%)	7 (11.3%)
vascR1*, *n* (%)	3 (4.8%)

*ICG* Indocyanine green, *R1* microscopic margins of specimen positive for tumor; *vascR1* microscopic presence of tumor on resection margins due to its detachment from a major vascular structure

*Calculated on the 62 patients undergoing liver resection form malignant disease only

The ICG administration for AIM-LF (LiMON test) was performed in 28 patients (36.8%) and was successful in the 85.7% of patients: the injection was repeated preoperatively in 4 patients after 6–8 h, due to a device reading error. The second attempt was successful and reliable in all cases. In 50% of cases, ICG administration was successful in obtaining both AIM-LF and AIM-TE and in 4 of the remaining patients, a further low dose of ICG (0.20–0.25 mg/kg) was administered 3–4 days before surgery to obtain tumor visualization (AIM-TE).

In 59 patients (77.6%), ICG was administered to achieve AIM-TE, and AIM-NS was performed in 19 cases (25%), AIM-PS in 7 (9.2%), AIM-VE in 16 patients (21.1%), and AIM-BE in 7 (9.2%). In liver resections for malignant indications, AIM-RM was performed in 25 cases (32.9%); and 21 (27.6%) and 12 patients (19.4%) underwent additional AIM-TEc and AIM-TEx, respectively. ICG fluorescence allowed the detection of 5 further histology-proven malignant lesions in addition to the ones identified preoperatively.

AIM-TE was successful in 56 out of 59 patients (94.9%). In 3 patients, all with HCC, superficial tumors could not be visualized as the ICG was retained in the liver for a prolonged period, likely due to the severity of the cirrhosis which caused a diffusely enhanced macronodular parenchyma, visible during the intraoperative examination. All 3 lesions were subsequently identified through IOUS. Five incidental lesions of more than 10 mm of diameter were detected through ICG fluorescence. The IOUS was performed in all cases and resulted suspicious for malignancy in 3 of them and were resected (2 cases) or ablated (1 case).

AIM-NS fluorescence onset was 10–20 s after systemic injection in all patients, but an incomplete staining was observed in 3 out of 19 cases (15.8%) due to unintentional partial segmental clamping.

AIM-PS fluorescence onset was 5–10 s after intra-pedicular injection. In 3 out of 7 patients (42.9%), a suboptimal positive staining was visualized due to poor diffusion of the dye throughout the afferent liver area.

The vascular enhancement onset during AIM-VE was 10–20 s after systemic administration and lasted between 30 and 75 s after each ICG bolus and was successful in 13 out of 16 patients 81.3% at the first attempt; in the remaining 3 cases, a second injection was necessary due to poor vascular staining. Overall, all procedures succeeded.

In 1 case out of 7 (14.3%), AIM-BE was not successful due to the liver parenchyma’s strong background signal and fluorescent lymphatic tissue rendering the interpretation of the biliary imaging as inconclusive.

In 2 patients out of 25 (8%) where AIM-RM had been performed, an increased resection margin was needed due to the presence of poorly vascularized parenchyma.

AIM-TEc and AIM TEx showed possible residual tumor on the cut surface in 4 out of 21 (19%) and 2 out of 12 (16.7%) lesions, respectively, mandating a further resection to enlarge the surgical margin which resulted in R0 resections in all 6 cases.

Patient characteristics are summarized in Table 2. Hepatopathy of various etiologies was present in 26 patients (34.2%). The most frequent preoperative diagnoses were hepatocellular carcinoma (HCC) in 26 patients (34.2%), colorectal liver metastases (CRLM) in 18 (23.7%), and cholangiocarcinoma (CCC) in 16 (21.1%), while 14 resections (18.4%) were performed for benign diseases. Forty-four patients (57.9%) had previous abdominal surgery, 9 of which (11.8%) were liver-directed procedures. In 43 cases (56.6%), a preoperative 3D rendering of the liver and the tumors with virtual simulations of the possible resection strategies and calculation of the future remnant liver volumes were performed. Preoperatively, a total of 117 lesions in 76 patients were diagnosed, with a median of one lesion per patient (0–6) and 2 patients with no lesions and intrahepatic biliary lithiasis. The most commonly performed liver resection was segmentectomy (in 20 patients, 26.3%), followed by bisegmentectomy and wedge resections, as shown in Table 3. The median IWATE score was 7 (3–12), with the 51.3% of cases characterized by an advanced or expert difficulty level (Table 3). Median surgical time was 295 min (75–800); median blood loss was 150 cc (10–2500 cc). Conversion was necessary in 10 cases (13.2%) due to severe adhesions (8 patients, 10.5%) and bleeding (2 patients, 2.6%). Seven conversions out of 10 (70%) happened in advanced or expert difficulty level IWATE score patients (8/39, 20.5%) while in 2 out of the 37 lower scores patients (5.4%).

**Table 2:** Preoperative characteristics of patients undergoing robotic liver resection and intraoperative indocyanine green fluorescence

Patients’ characteristics	Robotic liver resections *n*=76
Age, years	64.8 (20–84)
Sex (M), *n* (%)	47 (61.8%)
BMI, kg/m^2^	26.2 ± 4.1
ECOG/Karnofsky score	0 (1–2)/90 (50–100)
CHILD, *n* (%)*	
A5	17 (65.4%)
A6	9 (34.6%)
MELD*	8 (6–13)
Hepatopathy, *n* (%)	26 (34.2%)
Viral	16 (61.5%)
HCV	11 (68.7%)
HBV	5 (31.3%)
Alcoholic	7 (26.9%)
Metabolic	3 (11.5%)
Preoperative diagnosis, *n* (%)	
HCC	26 (34.2%)
CRLM	18 (23.7%)
CCC	16 (21.1%)
Benign	14 (18.4%)
Intrahepatic biliary lithiasis	2 (2.6%)
Preoperative number of lesions	117
Lesions per patients	1 (0–6)**
Malignant, *n* (%)	102 (87.2%)
ASA score	3 (1–3)
Charlson comorbidity index	5.7 ± 2.8
Platelets (×10^3^ U/L)	195 (68–559)
INR	1.05 (0.89–1.3)
Total bilirubin (mg/dL)	0.62 (0.10–2.59)
Albumin (g/dL)	4.2 ± 0.55
Creatinine (mg/dL)	0.85 (0.59–1.58)
Previous abdominal surgery, *n* (%)	44 (57.9%)
Previous liver surgery, *n* (%)	9 (11.8%)
Previous chemotherapy, *n* (%)°	16 (21.1%)

*BMI* Body mass index; *ECOG* Eastern Cooperative Oncology Group; *MELD* model for end-stage liver disease; *HCC* hepatocellular carcinoma; *CRLM* colorectal liver metastases; *CCC* cholangiocarcinoma; *ASA* American Society of Anesthesiologists; *INR* international normalized ratio

*Calculated on 21 patients with hepatopathy

**Calculated on 59 patients, as two had no lesions and underwent liver resection for intrahepatic lithiasis

°Calculated on malignant indications only

**Table 3:** Intraoperative characteristics of the robotic liver resections

Surgery characteristics	Robotic liver resections *n*=76
Type of resection, *n* (%)	
Segmentectomy	20 (26.3%)
Bisegmentectomy	15 (19.7%)
Subsegmentectomy/wedge resection	14 (18.4%)
Left hepatectomy	9 (11.8%)
Right hepatectomy	6 (7.9%)
Extended right/left hepatectomy	2 (2.6%)
RALPPS	2 (2.6%)
Other	8 (10.5%)
Postero-superior segments, *n* (%)	37 (48.7%)
Iwate score, median (range)	7 (3–12)
Difficulty level, *n* (%)	
Low	5 (6.6%)
Intermediate	32 (42.1%)
Advanced	31 (40.8%)
Expert	8 (10.5%)
Associated thermal ablations, *n* pts (%)	18 (23.7%)
Surgical time, min	295 (75–800)
Intraoperative blood loss, mL	150 (10–2500)
Intraoperative blood transfusions, *n* (%)	13 (17.1%)
Pringle maneuver, *n* (%)	51 (67.1%)
Pringle maneuver time, min	22.8 (5–80)
Open conversion, *n* (%)	10 (13.2%)

*RALPPS* Radiofrequency-assisted liver partition with portal vein ligation for staged hepatectomy

Overall postoperative complications occurred in 19 cases (25%) (Table 4), of which 8 (10.5%) were Clavien–Dindo grade ≥3: 4 patients (5.3%) developed a biliary leak requiring endoscopic retrograde cholangiopancreatography (ERCP), 4 patients (5.3%) a perihepatic collection, and 2 patients (2.6%) a pleural effusion both requiring a percutaneous drainage. No in-hospital or 90-day mortality was observed. Median length of stay was 5 days (2–27 days).

**Table 4:** Postoperative outcomes and complications of patients undergoing robotic liver resection and intraoperative indocyanine green fluorescence

Outcomes	Robotic liver resections *n*=76
ICU stay, days	1 (0–4)
Postoperative transfusions, patients *n* (%)	3 (3.9%)
Postoperative complications, patients *n* (%)*	19 (25%)
Clavien-Dindo <3, patients *n* (%)	15 (19.7%)
Ascites Collection	9 (11.8%) 6 (7.9%)
Pleural Effusion	4 (5.3%)
Anemia	3 (3.9%)
Pneumonia	2 (2.6%)
Other	3 (3.9%)
Clavien-Dindo ≥3, patients *n* (%)	8 (10.5%)
Biliary fistula	4 (5.3%)
Abdominal collection requiring drainage	4 (5.3%)
Pleural drain	2 (2.6%)
Comprehensive complication index	8.7 (8.7–44.9)
Hospital length of stay, days	5 (2–27)

*ICU* Intensive care unit

*Some patients developed more than one complication

## Discussion

Robotic surgery and ICG administration are highly versatile and complementary tools that can enable technical improvements in many settings [38–41], particularly in hepatobiliary surgery [16, 21, 22]. ICG fluorescence visualization is affected by many factors, and, before routinely incorporating this technique into surgical practice, adequate training and experience must be developed. Moreover, there remains some uncertainty regarding the optimal ICG dosing strategy in the context of the Da Vinci® robotic platform, which is a powerful visualization system [42]. Strong signals could be due to an excessive dose, metabolic disorders, fluorescence dispersion, or poor team’s coordination. In contrast, slow ICG injection, insufficient doses, excessive camera distance, and incorrect dye flushing after administration could lead to poor visualization [16, 18, 43]. However, our series of RLR performed with the aid of ICG administration proves that its implementation and routine use can provide a most useful aid in diverse settings.

### AIM-LF (LiMON test)

Since the 1980s, ICG has been used to assess the liver function prior to hepatobiliary surgery [44]. Generally, we consider as acceptable thresholds for a safe major hepatectomy a plasma disappearance rate (PDR) >18% per minute and a retention ratio after 15 min (R15) <10% [45], evaluated in combination with the future liver remnant volume calculated on 3D rendering models. However, the use of LiMON test as a combined functional and tumor enhancement test (AIM-LF + AIM-TE) has been scarcely described in detail [18, 46, 47]. If a LiMON test has been performed in the 3–7 days prior to the surgical resection of liver tumors, a further ICG injection can be deferred, considering that primary and secondary liver tumors can retain ICG and be visible for up to 14 days after administration [18, 46]. It needs to be considered that CRLM can show some erratic enhancement with different patterns that can also be linked to the tumor grading and differentiation [15, 46, 48]. To avoid low enhancement, a further ICG administration can be safely and effectively repeated if a functionality test has been performed earlier than 7 days before surgery; however, a reduced dose (0.2 mg/kg) may be recommended in such a scenario [46].

We performed a LiMON test mainly prior to major hepatectomies, to assess the liver function and simultaneously, when possible, exploit its intraoperative fluorescence properties. Occasionally, as seen in our series, the functionality test may fail due to a fault in the reading and/or low patient oxygen saturations; in this case, the test can be safely repeated on the same day. In our experience, surgical timing is another factor that can hamper the visualization of malignant tumors, and a preoperative, lower-dose ICG injection is recommended if surgery is delayed, especially in non-cirrhotic patients. We usually perform a LiMON test 3–4 days prior to surgery in non-cirrhotic patients and 5–6 days in cirrhotic patients. We administer an additional reduced dose of 0.2 mg/kg if surgery is delayed (>4 days in non-cirrhotic patients, >6 days in cirrhotic patients). In our experience, we consider the LiMON test to be a useful adjunct even in settings where its results have not been fully validated, such as in cases of non-cirrhotic liver parenchyma and severe cholestasis.

### Tumor enhancement (AIM-TE), Fig. 1

The dose and timing of preoperative ICG administration for tumor enhancement purposes have long been a topic of discussion. The ideal timing lies anywhere from 12 h to 14 days before surgery [16, 46, 48–50], with a preference for 24–72 h preoperatively [16, 46, 51–53]; and with a dose of 0.2–0.5 mg/kg considered as optimal. In our experience, lower doses and an administration timing between 48 and 72 h preoperatively provided the best results, possibly thanks to the elimination of some patient-specific variables (such as cardiac function, blood pressure, body fat distribution, and metabolic disorders), as previously reported [16, 21]. The reported accuracy rate varies at around 85–93% [18, 21, 46, 51, 53].

**Fig. 1:**
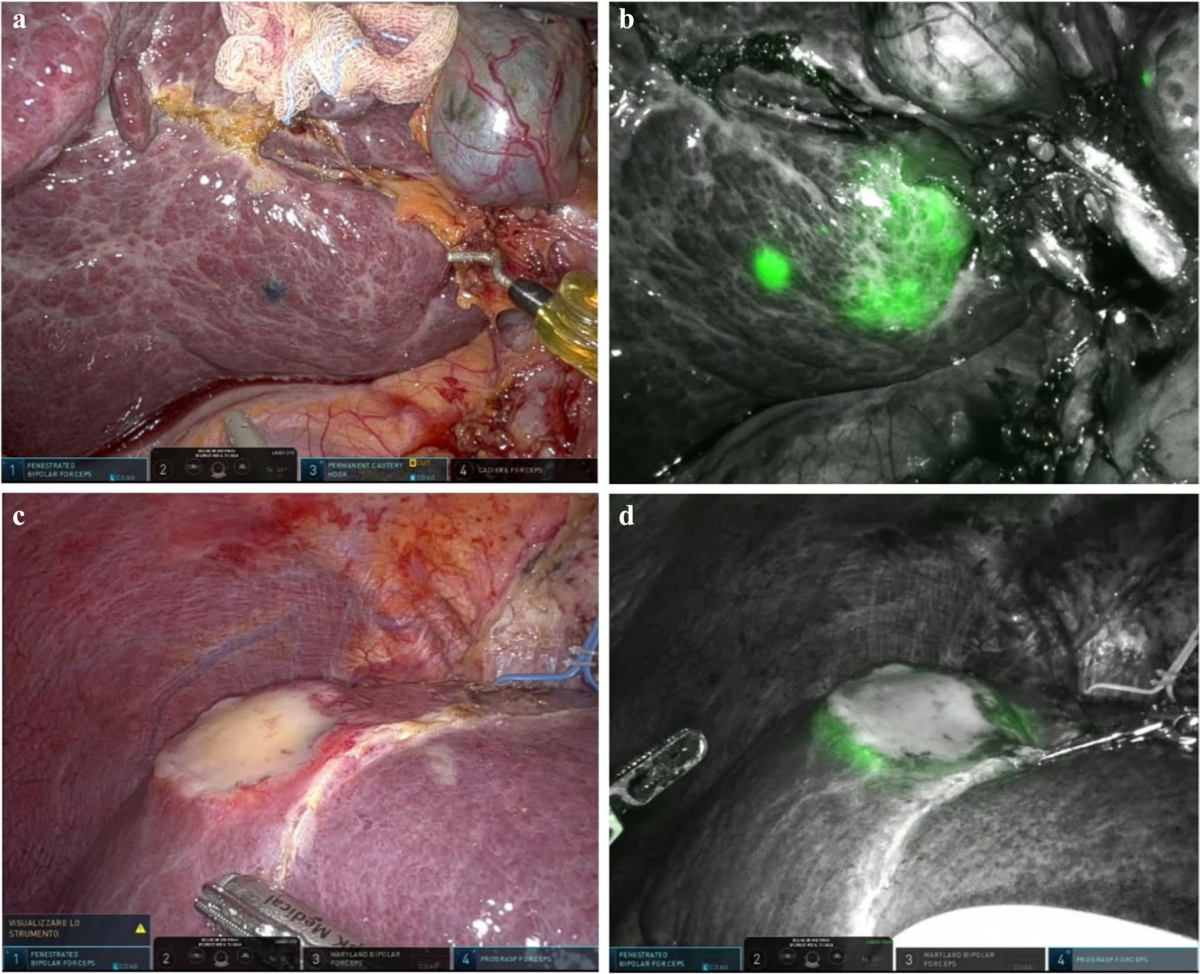
Indocyanine green fluorescence visualization for tumor identification purposes (AIM-TE) of a hepatocellular carcinoma (HCC) and a colorectal liver metastasis (CRLM). **a** A 68-year-old patient with HCV-related cirrhosis a SgVI HCC. **b** The lesion becomes visible only through indocyanine green fluorescence. To note how a small biliary cyst close to the HCC nodule becomes highly fluorescent. **c** A 57-year-old patient with a 4.5 cm, SgIVa CRLM. **d** The lesion was primarily constituted by desmoplastic and necrotic tissue, and at fluorescence showed the typical ring enhancement, due to peripheral biliary compression and delayed bile excretion

Liver fibrosis, cirrhosis, bile excretion disorders, and previous extensive chemotherapy complicate dose and timing calculation. In these cases, it is advisable to reduce doses and/or prolong the interval timing, as in these specific scenarios, there is an increased false positive rate, reaching peaks of 40% [16, 43, 46, 52]. These enhanced spots often show up as small superficial areas of fluorescence (1–2 mm), due to increased biliary retention in the absence of malignancy. In cirrhotic patients, an interval of up to 7 days should be respected [54], and ICG administration should rarely be performed closer than 48h prior to surgery [18]. However, no clear cut-off has been established to date, and a patient-specific evaluation should always be advocated. ICG fluorescence limitations in this setting are the low penetration depth (around 8 mm) and its poor efficacy in visualizing deep lesions [16].

We routinely administer 0.25–0.50 mg/kg ICG, 2–4 days before liver surgery for HCC [15, 16]. In cirrhotic patients, since the retention time tends to be increased, we administer a lower dose (0.25–0.30 mg/kg) 3–4 days before surgery, or an intermediate dose (0.30–0.40 mg/kg) in case of delayed surgery (5–6 days). For CRLM and CCC patients, the dose is usually intermediate/high (0.30–0.50 mg/kg). It needs to be considered that CCC lesions can show the presence of extensive necrotic/desmoplastic tissue, compromising an optimal ICG fluorescence visualization.

### Negative staining (AIM-NS), Fig. 2

Negative staining has been previously described as an advantageous method to reach optimal resection margins when performing anatomical resections, even in the living donor liver transplantation setting [22, 47, 53, 55, 56]. The Glissonean approach by following the first and second order portal branches may facilitate minimally invasive liver surgery by allowing rigorous anatomical resections [57]. In this setting, the intraoperative ICG infusion following the target pedicle identification aimed at achieving a negative or positive staining, allows a clearer identification of the anatomical landmarks and eventually leads to more precise and safer lobar, segmental, or subsegmental resections.

**Fig. 2:**
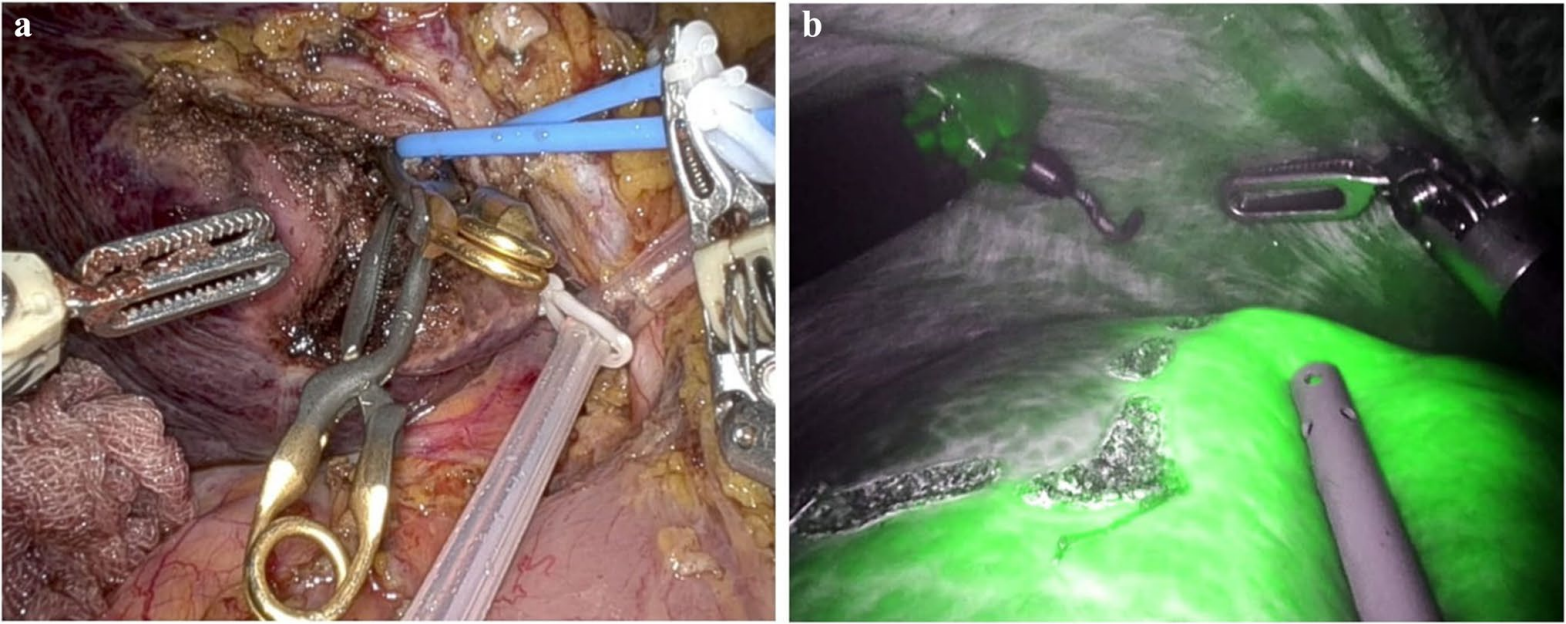
Indocyanine green fluorescence visualization for negative staining (AIM-NS) on a 68-year-old patient with HCV-related cirrhosis and bifocal HCC undergoing SgVII anatomical resection. **a** SgVII pedicle is identified via intraoperative ultrasound and, after hepatotomy, it is dissected, encircled with a vessel loop, and clamped with bulldogs. **b** After the systemic injection of an ICG bolus of 2.5 mg, the future liver remnant is enhanced, and the right posterior sector’s limits are marked with the cautery hook

The intravenous ICG dose has been reported to vary from 1.25 to 2.5 mg, injected intravenously after clamping the targeted segment [22, 47, 53, 55, 56]. Complete visualization requires approximately 3 min after administration and, when correctly performed, results in a very high success rate [56]. However, several factors could hamper optimal staining, such as the presence of multiple portal branches supplying the target anatomical territory, or a collateral circulation (such as an accessory left hepatic artery, inferior phrenic artery or other anatomic vascular variations), and technical mistakes such as the clamping of a wrong portal pedicle [47]. It is our routine practice to check for the discoloration of the liver parenchyma after the pedicle clamping, in conjunction with IOUS Doppler evaluation prior to the ICG injection, in order to reduce the chance of accidental staining of the incorrect liver areas that could jeopardize the fluorescence result. If negative staining is unsuccessful, it is advisable to suspend ICG visualization and continue the hepatic resection with the guidance of other tools, since reversing an inaccurate staining is not possible and the incorrect fluorescence will be maintained for several hours [19, 22, 32, 47].

To achieve AIM-NS, we systemically inject 2.5 mg of ICG in 2 mL of injectable solution, after the clamping or division of the target segment(s) portal inflow. Generally, the fluorescence onset is 10–20 s post-administration and remains for the duration of surgery.

### Positive staining (AIM-PS), Fig. 3

Positive staining has been reported less frequently in the literature, possibly due to an increased technical difficulty [18, 47, 58, 59], leading to a lower efficacy rate compared to negative staining [22]. ICG dilution can vary from 0.005 to 0.025 mg/ml, injected in 1 to 10 ml of injectable solution, depending on the size of the target liver parenchyma [18, 47, 58].

**Fig. 3:**
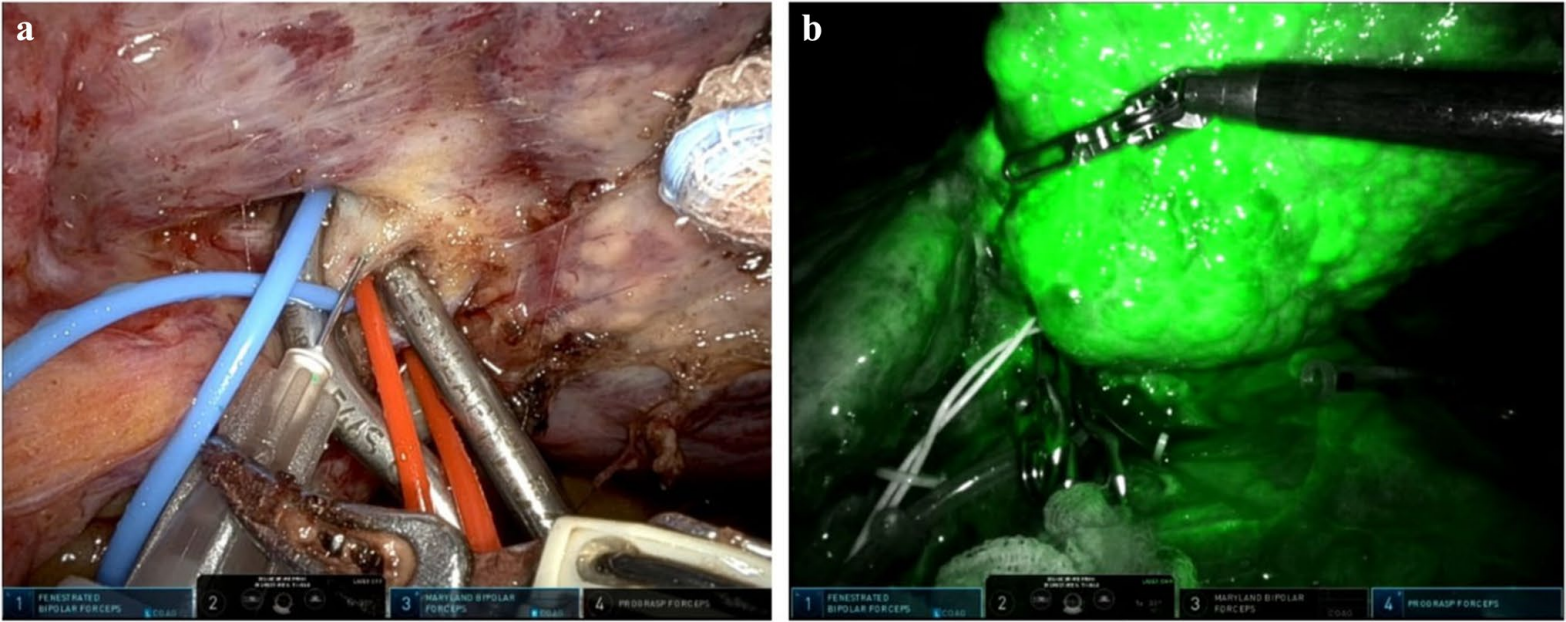
Indocyanine green fluorescence visualization for positive staining (AIM-PS) on a 65-year-old patient undergoing a left lateral hepatectomy for an HCC on HBV-related cirrhosis. **a** The pedicle for SgII-III is encircled with two vessel loops (blue for the portal pedicle and red for the artery), and clamper with bulldogs. **b** An ICG bolus of 1.25 mg is injected in the portal branch distal to the clamps to obtain the positive staining

Aoki and colleagues described the preoperative percutaneous injection of ICG dye into the target pedicles through an 18-gauge percutaneous transhepatic cholangio-drainage-needle under US guidance, reporting an 86% success rate [58]. However, in our experience, direct intraoperative injection after the required liver mobilization and the selective pedicle identification and isolation is easier, and further facilitated by the seven degrees of freedom granted by robotic instruments.

Ueno and colleagues described a combined technique with interventional radiology in a hybrid operating room [59]. However, this approach presents many technical limitations (increased costs, time-consuming, absence of a hybrid operating room in many hospitals and challenging spatial integration with the robotic system).

When positive staining is performed intraoperatively, its success is variable and generally reported at around 50% [47], similar to the rate observed in our series (57.1%). Failure to inject in the correct targeted portal branch, technical difficulties with surgical instruments, and retrograde blood flow could lead to the enhancement of undesired segments or incomplete staining [47]. Generally, to test the accuracy of pedicle clamping prior to ICG injection, it is advisable to rely on an accurate IOUS exploration with Doppler imaging. As in negative staining, correcting a poor demarcation after a positive staining attempt is rarely possible, and other devices and/or techniques must be used to complete the resection [19, 22, 32, 47].

We usually administer 1.25 mg of ICG in 5 mL of injectable solution in the portal pedicle directly downstream of the clamp. Fluorescence onset is almost immediate (5–10 s). Generally, it is not necessary to increase the injected volume or the ICG dose if enhancement of a larger area is required.

### Vessels’ identification (AIM-VE), Fig. 4

Despite being widely used in other surgical disciplines [38–40], the literature on ICG fluorescence in hepatobiliary surgery for vessel enhancement and identification is scarce [60]. An early experimental porcine model demonstrated how intravenous ICG injection sequentially enhanced all intrahepatic and extrahepatic anatomic structures (the hepatic and cystic arteries after 10 s, the liver after 10 min, gallbladder and bile ducts after 40 min, and duodenum after 60 min) [61]. In humans, the most frequently reported use of ICG for this purpose is after liver transplantation; ICG can be administered intravenously after portal vein and hepatic artery reconstruction (at a dose of 3.75 mg) [60]. In our series, we were successful in obtaining satisfactory vessels enhancement in all 16 patients, although in 3 of them required a second ICG administration.

**Fig. 4:**
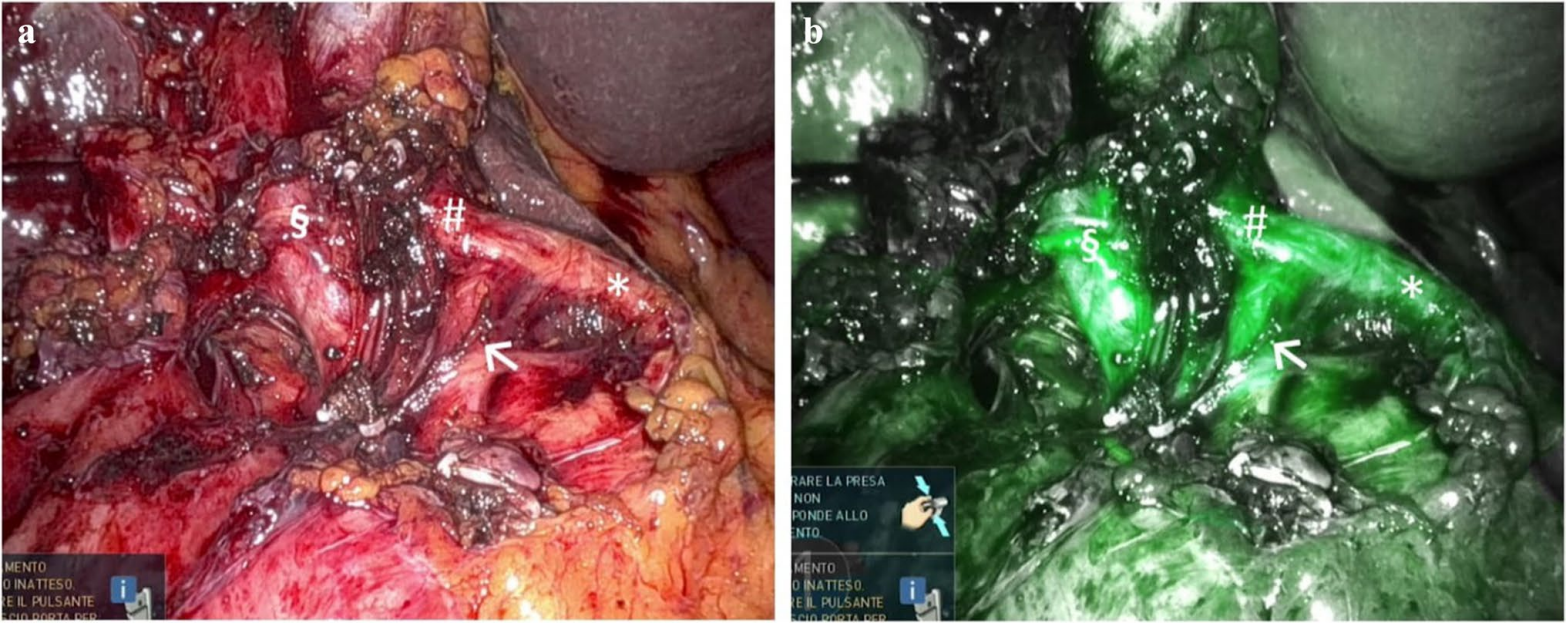
Indocyanine green fluorescence visualization for vessels’ identification (AIM-VE) on a 73-year-old patient undergoing wedge resection of SgV-VI for a T2 gallbladder carcinoma. In order to perform a safe hepatic hilar lymphadenectomy, 2.5 mg of indocyanine green are administered in order to visualize the vascular hilar elements: common hepatic artery (*), gastroduodenal artery (→), left hepatic artery (#), and right hepatic artery originating from the superior mesenteric artery (§). **a**: pre ICG administration, **b**: post ICG administration

ICG is injected as per AIM-NS (2.5 mg in 2 mL) and follows the same time of onset (10–20 s). In the case of failure or ambiguous interpretation, it can be re-administered once it had cleared. However, a delayed parenchymal and biliary staining must be considered and could hamper further assessments.

### Biliary tree enhancement (AIM-BE), Fig. 5

Intuitively, the first described application of ICG fluorescence for biliary tract visualization was during laparoscopic cholecystectomies [62]. More recently, its applications widened to other procedures such as hepatectomies, living donor hepatectomy, hepatic pedicle lymphadenectomy, and hilar dissection in complex and high-risk settings (previous pancreatitis, biliary stenting, recurrent cholangitis, multi-operated patients) [16, 63–65]. This technique allows a safer dissection of the critical structures and the identification of eventual abnormal anatomy or accessory biliary ducts [16]. A recent randomized controlled clinical trial proved that ICG cholangiography was more sensitive in visualizing extrahepatic biliary structures than white light visualization alone [66], and was superior over saline solution [67]. This technique’s limitation is the lack of distal biliary tract visualization (due to the surrounding pancreatic parenchyma) and the poor visualization of stones in the common bile duct [16]. The two main techniques to reach satisfactory biliary enhancement are preoperative intravenous injection and intraoperative intrabiliary administration.

**Fig. 5:**
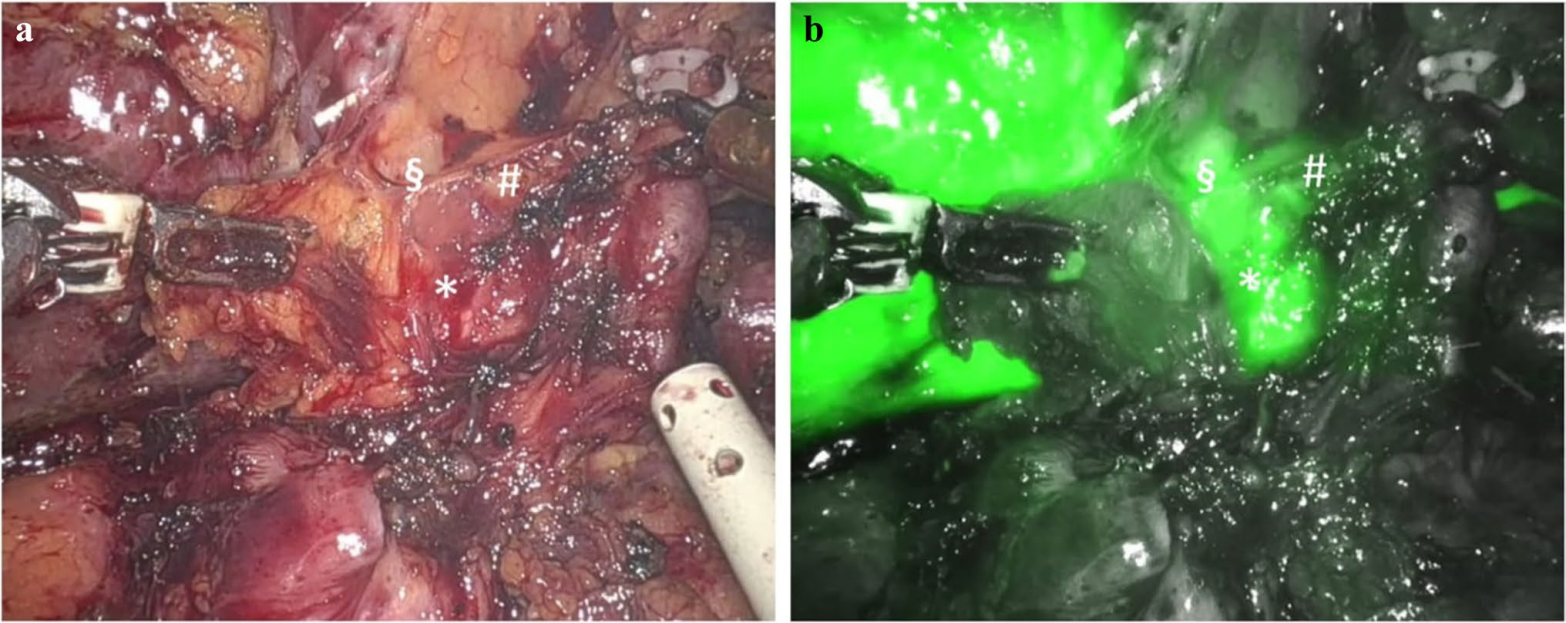
Indocyanine green fluorescence visualization for biliary tree enhancement (AIM-BE) in a 68-year-old patient undergoing left hepatectomy for a hilar cholangiocarcinoma. At the anesthesia induction, 2.5 mg of indocyanine green are administered systemically. **a** Without fluorescence, the bile ducts appear scarcely visible due to the presence of lymphatic and inflammatory tissue. **b** Fluorescence visualization allows a better visualization of the main bile duct (*) as well as the left (#) and right (§) ducts

Intravenous injection is performed 30–60 min before surgery [48, 61] or 15–30 min before dissection of the hilar plate in a transplantation setting [64], at a dose of 2.5 mg or 0.05 mg/kg [16, 48, 65]. Residual biliary fluorescence has been reported with preoperative administrations up to 24 h prior to surgery [63]. The advantages of this technique over conventional radiographic intraoperative cholangiography are the lack of ionizing radiation, its time-saving execution, the fluorescence duration, and the need of less staff and equipment in the operating room (i.e., radiology technician, c-arm) [63, 68].

Intrabiliary administration is mainly performed through the cystic duct (after common bile duct clamping) with variable reported concentrations (0.025–0.5 mg/mL) depending on the purpose and location of the biliary ducts of interest [48, 66, 67, 69]. This method is generally preferred when performing a biliary leak test at the end of the liver resection [64, 67, 69].

We usually inject an ICG bolus of 2.5 mg in 2 mL of injectable solution either prior to anesthesia induction or in the early stages of surgery to perform the biliary assessment 45–60 min later. In our experience, a more prolonged timing compared to that described by other reports [48, 61, 64], allows for some ICG liver excretion and lessens the background parenchymal glare that can compromise an accurate visualization, as observed in the unsuccessful case of our series.

### Liver cut surface vascularization (AIM-RM), Fig. 6

**Fig. 6:**
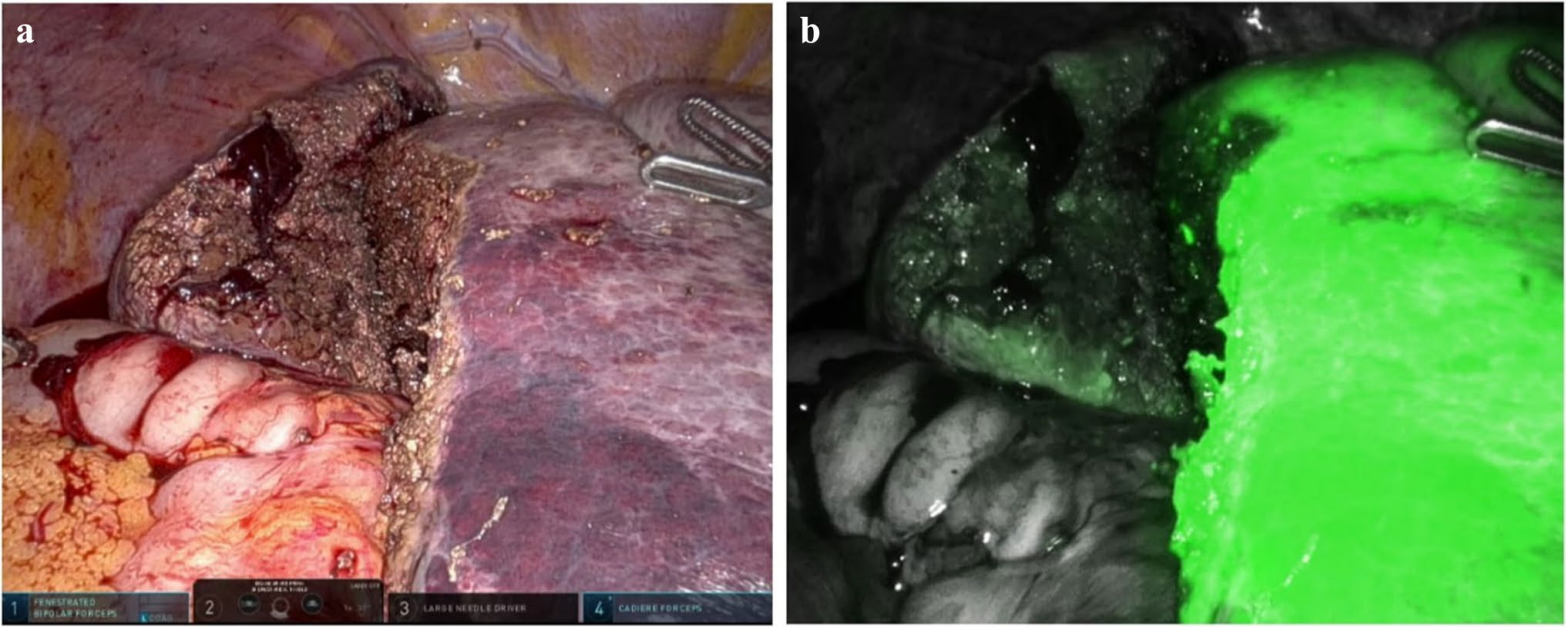
Indocyanine green fluorescence visualization of the liver cut surface perfusion (AIM-RM) after the end of a SgVI-VII bisegmentectomy for HCC in a 73-year-old patient. **a** The remnant liver’s color appears macroscopically poorly perfused. **b** After systemic injection of 2.5 mg of indocyanine green, the resection shear results completely enhanced, indicating a good vascularization of the liver remnant without the need for resection enlargement

Following RLR, the adequacy of the blood supply to the remnant liver can effectively be assessed with an intravenous ICG injection at the end of the parenchymal transection [53, 55]. Literature on this topic is scarce, and, to the best of our knowledge, no report has systematically this indication. We did not observe a direct correlation between the macroscopic appearance of the liver surface and its adequate vascularization, leading to a resection enlargement following AIM-RM in 2.6% of cases, and preserving the parenchyma in case of satisfactory fluorescence regardless of the macroscopic appearance, as in Fig. 6.

To achieve AIM-RM, we systemically inject 2.5 mg ICG in 2 mL of injectable solution. Generally, the fluorescence onset is 10–20 s post-administration and lingers for the whole duration of surgery. Intuitively, this assessment is not feasible if the parenchyma is still retaining ICG because of a recent administration (i.e., AIM-NS).

### Residual liver cut surface tumor identification (AIM-TEc) and ex-situ specimen assessment (AIM-TEx), Fig. 7

After the completion of RLR, ICG fluorescence can be used to detect residual tumor on the cut surface. A few reports describe the application of fluorescence both on liver cut surface [53] and on the specimen [46, 53], allowing a resection enlargement with greater margins when appropriate.

**Fig. 7:**
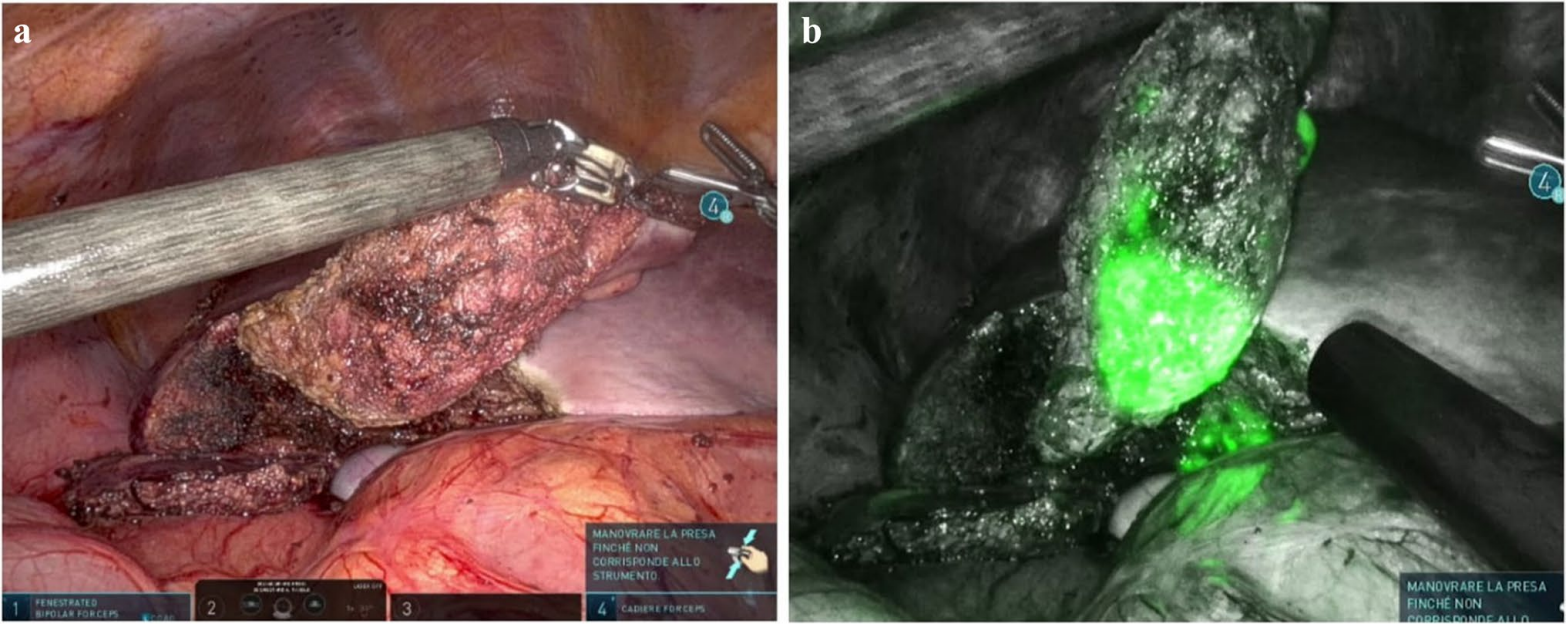
Indocyanine green fluorescence for liver resection margin evaluation on the cut surface and specimen (AIM-TEc and AIM-TEx) after a wedge SgV-VI resection of an HCC in a 57-year-old patient affected by HCV-related cirrhosis. The fluorescence visualization (**b**) shows tumoral tissue surfacing on the specimen as well as a fluorescent area in the remnant liver resection margin, not visible macroscopically or through ultrasound examination (**a**), mandating a resection enlargement

Since these techniques are mostly coupled with other aims, they usually do not require additional administrations and dose and timing vary depending on the main enhancing method. These aims could be applied either after AIM-LF and AIM-TE. Liver staining techniques do not usually combine well with AIM-TEc/AIM-TEx, since spots of enhanced liver parenchyma on the resection plane could be residuals from healthy unresected parenchyma (after AIM-NS) or the cut surface should appear completely enhanced (after AIM-PS). AIM-BE and AIM-VE combine poorly with AIM-TEc/AIM-TEx due to the almost homogeneous fluorescence of the whole liver parenchyma that follows their application, as would happen in case of AIM-RM, which can be performed in combination with AIM-TEc/AIM-TEx but only afterwards. An immediate, easy to perform and reliable assessment of the resection margins that can directly translate into a benefit for the patients by reducing the R1 rate, is a useful tool that allowed additional 4 patients (6.5%) of our series to have a radical resection.

Patients undergoing liver resections can often benefit from more than one ICG administration during the perioperative course, and more than one aim can be achieved before and during surgery, via a single or multiple injections. This is made possible by the very low toxicity rate of ICG and, most of all, by an accurate, patient-specific preoperative planning process. A preoperative ICG administration, if performed with adequate dose and timing, can allow a repeated intraoperative dose to obtain vessels or anatomical areas identification or transection margin perfusion evaluation. However, care must be taken not to combine techniques that could invalidate each other’s results, such as an ICG injection for vessel identification that could result in a homogeneous parenchyma fluorescence that will hamper a subsequent positive or negative staining, if needed. Experience, team coordination, and accurate planning are essential requirements to obtain successful results. In all described cases the Intuitive® Firefly near-infrared camera was used, being the only one available at present for robotic systems, in contrast to open and laparoscopic devices [70].

**Fig. 8:**
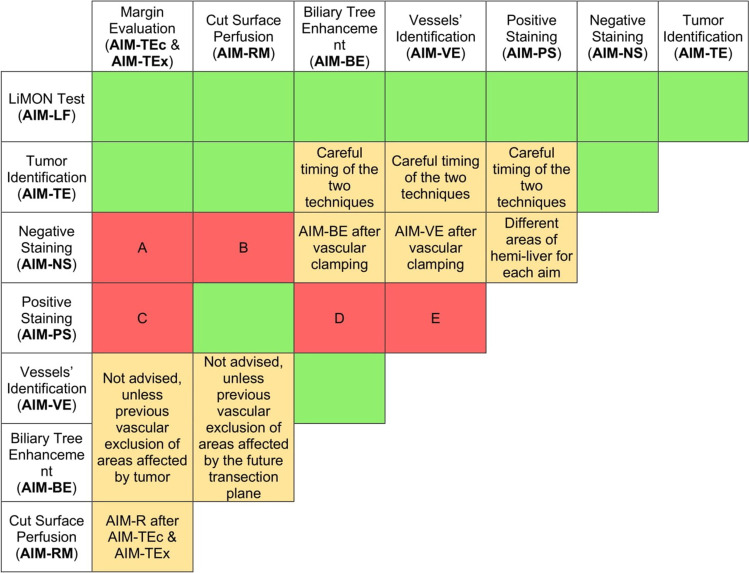
Timing and compatibility of Indocyanine Green fluorescence techniques. Red cells symbolize scarce/absent compatibility, yellow cells conditional compatibility. **A** At the end of a negative staining-guided transection, residual enhanced spots on the cut surface could be expression of residual unresected healthy parenchyma. **B** At the end of a negative staining-guided transection, the remnant liver is already completely enhanced, causing a new ICG injection for the cut surface perfusion to be ineffective. **C** At the end of a positive staining-guided transection, an optimal resection shear should be not enhanced but with possible residual positive-stained healthy parenchyma areas, non-distinguishable from residual tumor. **D** Biliary visualization affects all liver parenchyma surrounding the enhanced positively stained area. **E** Vascular visualization affects all liver parenchyma surrounding the enhanced positively stained area

**Fig. 9:**
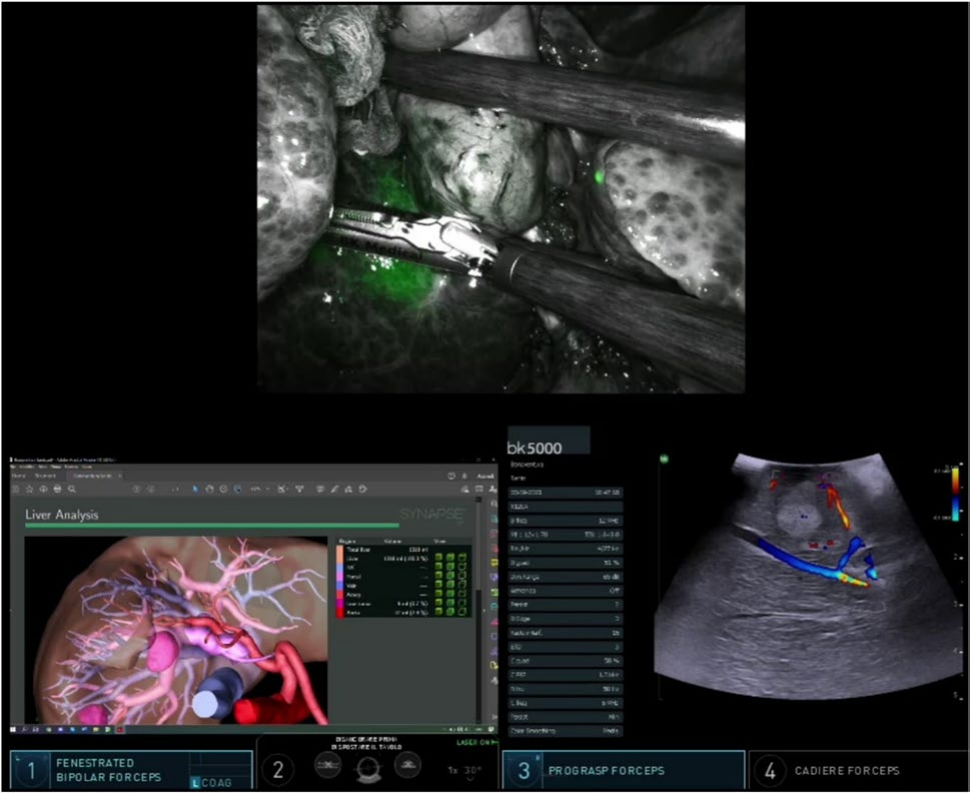
The integrated robotic platform permits the simultaneous visualization of several systems together with the operating field, such as radiologic images, 3D reconstructions, and intraoperative ultrasounds. This function can reduce workflow disruption and provide instant comparison between multiple modalities

A complete guide of timing and compatibility of the techniques set out in this paper is shown in Fig. 8. Despite being based on prospectively collected data, our study is limited by being undertaken at a single center and with a relatively small sample size lacking a control group to adequately appreciate the effectiveness of ICG administration in all the explored areas. Our results show that ICG fluorescence in RLR has high success rates, with the preoperative administration finalized at the tumor enhancement (AIM-TE) representing the most common versatile utilization of this technology, and the positive staining (AIM-PS) representing the most challenging one. A possible explanation of the lower success rates of the positive staining of our series could be related to the higher technical complexity and the upstream vascular clamping that does not allow an ideal blood flow necessary to the adequate distribution of the ICG. The integration of ICG fluorescence with IOUS and 3D models in the robotic platform with their simultaneous visualization (Fig. 9) represents a breakthrough innovation that allows to maximize the surgeon’s spatial awareness and to overcome most of the drawbacks of the robotic technique, resulting in enhanced safety and efficacy.

In our experience, ICG fluorescence has proven to be a valuable tool in hepatobiliary surgery and allows several diverse assessments according to each individual patient and condition-specific goals and issues. RLR can greatly benefit from the routine integration of ICG fluoresce evaluation in all aspects of liver functional assessment, anatomical and vascular evaluation, tumor detection, and resection margins assessment.

## Abbreviations

AIM-BE: Biliary structures’ enhancement via indocyanine green fluorescence
AIM-LF: Liver function assessment test via indocyanine green administration
AIM-NS: Negative staining via indocyanine green fluorescence
AIM-PS: Positive staining via indocyanine green fluorescence
AIM-RM: Evaluation of resection margin vascularization via indocyanine green fluorescence
AIM-TE: Preoperative indocyanine green administration for malignant lesions’ enhancement
AIM-Tec: Malignant lesions’ enhancement on the cut surface via indocyanine green fluorescence
AIM-TEx: Ex situ assessment on the specimen via indocyanine green fluorescence
AIM-VE: Vascular structures’ enhancement via indocyanine green fluorescence
ASA: American Society of Anesthesiologists
BMI: Body mass index
CCC: Cholangiocarcinoma
CCI: Charlson comorbidity index
CEUS: Contrast-enhanced abdominal ultrasound
CT: Computed tomography
CRLM: Colorectal liver metastases
ECOG: Eastern cooperative oncology group
HCC: Hepatocellular carcinoma
ICG: Indocyanine green
ICG-R15: Retention rate at 15 min after indocyanine green intravenous administration
INR: International normalized ratio
IOUS: Intraoperative ultrasound
ISGLS: International study group of liver surgery
MELD: Model for end-stage liver disease
MRI: Liver gadoxetic acid–enhanced magnetic resonance imaging
PDR: Plasmatic disappearance rate
PET: Positron-emission tomography
PHLF: Post-hepatectomy liver failure
R0: Resection margin free from malignant cells (distance ≥1 mm)
R1: Presence of malignant cells on the resection margin (distance <1 mm)
RLR: Robotic liver resections
